# Supplementary material for: Divergent subregional information processing in mouse prefrontal cortex during working memory
Source: Commun Biol. 2024 Oct 1;7:1235. doi: 10.1038/s42003-024-06926-8 (PMC11445572; doi:10.1038/s42003-024-06926-8)
Supplement: Supplementary file 2 — Supplementary Information [file 42003_2024_6926_MOESM2_ESM.pdf]

Supplementary Information

Divergent Subregional Information Processing in Mouse Prefrontal Cortex during Working Memory

Alex Sonneborn[1], Lowell Bartlett[1], Randall J. Olson[1], Russell Milton[1], Atheir I. Abbas[1,2,3*]

[1]Department of Behavioral Neuroscience, Oregon Health and Science University, Portland, OR 97239

[2]Department of Psychiatry, Oregon Health and Science University, Sam Jackson Hall, Portland, OR, 97239

[3]VA Portland Health Care System, 3710 SW US Veterans Hospital Rd, Portland, OR 97239

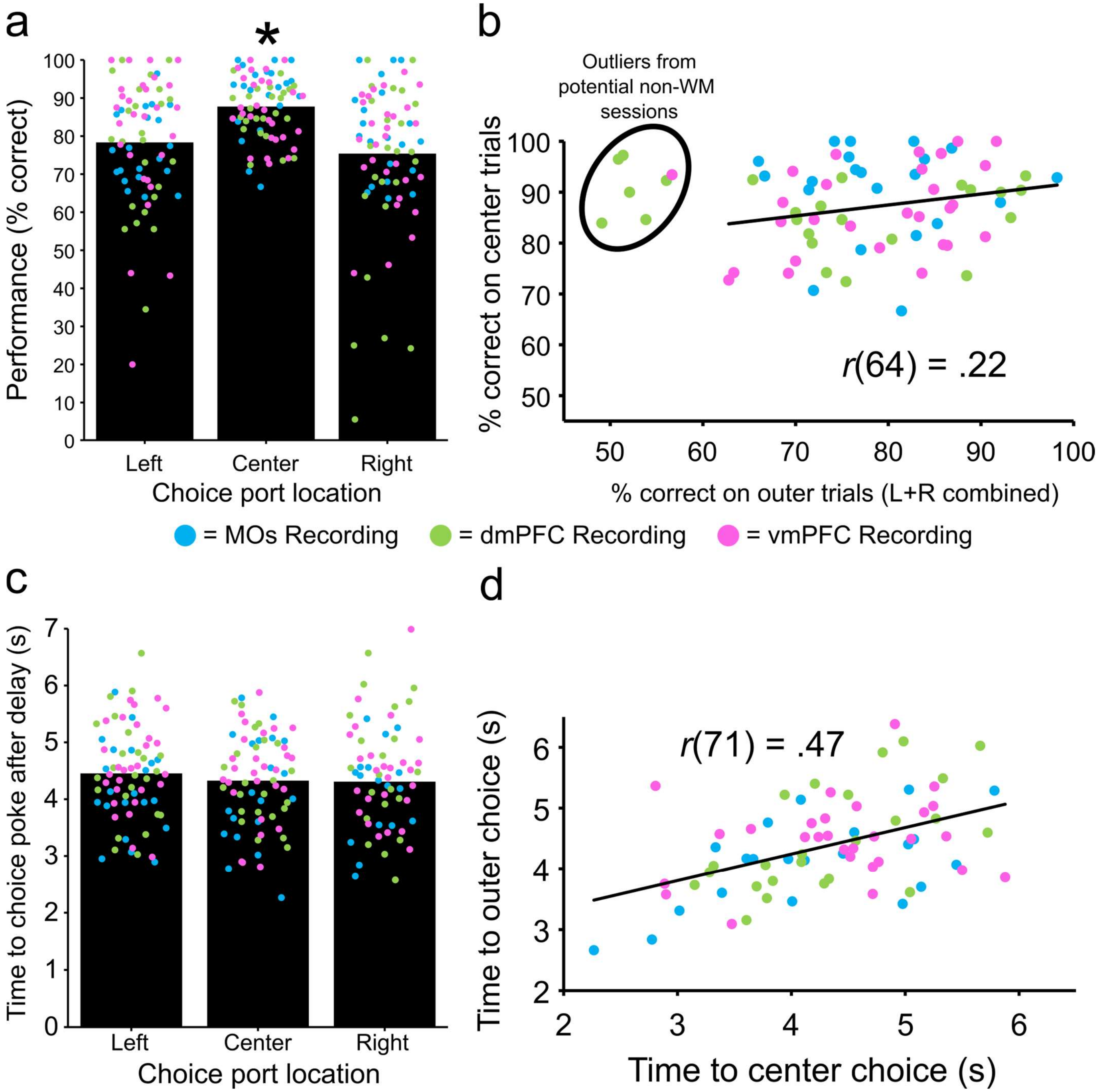

Supplementary Fig. 1: Trial-type specific behavioral analyses organized by session. a Trial-type specific performance combining all sessions from all three groups. Each bar indicates the average performance on trials when the choice port was either Left, Center, or Right and each color-coded dot represents combined performance on an individual session. Mice perform significantly better on trials in which the center port is the choice port (Kruskal-Wallis H test, $\chi^2(2) = 24.64$, $p = 4.5e\text{-}6$). **b** Correlation between performance on outer (Left and Right choice trials combined) and center trials. Correlation was run on the 66 sessions after removal of the potential outlier sessions in which mice were likely not using a working memory strategy to complete the task (inside of black oval). Interestingly, the six dmPFC sessions in this group all came from the same mouse (dmPFC 4). r value and degrees of freedom are listed on this panel and panel d. **c** Average length of time, in seconds, that it takes mice to make a choice after the delay period ends for Left, Center, and Right trials. **d** Correlation between time to the choice poke from the end of the delay on center trials vs outer trials.

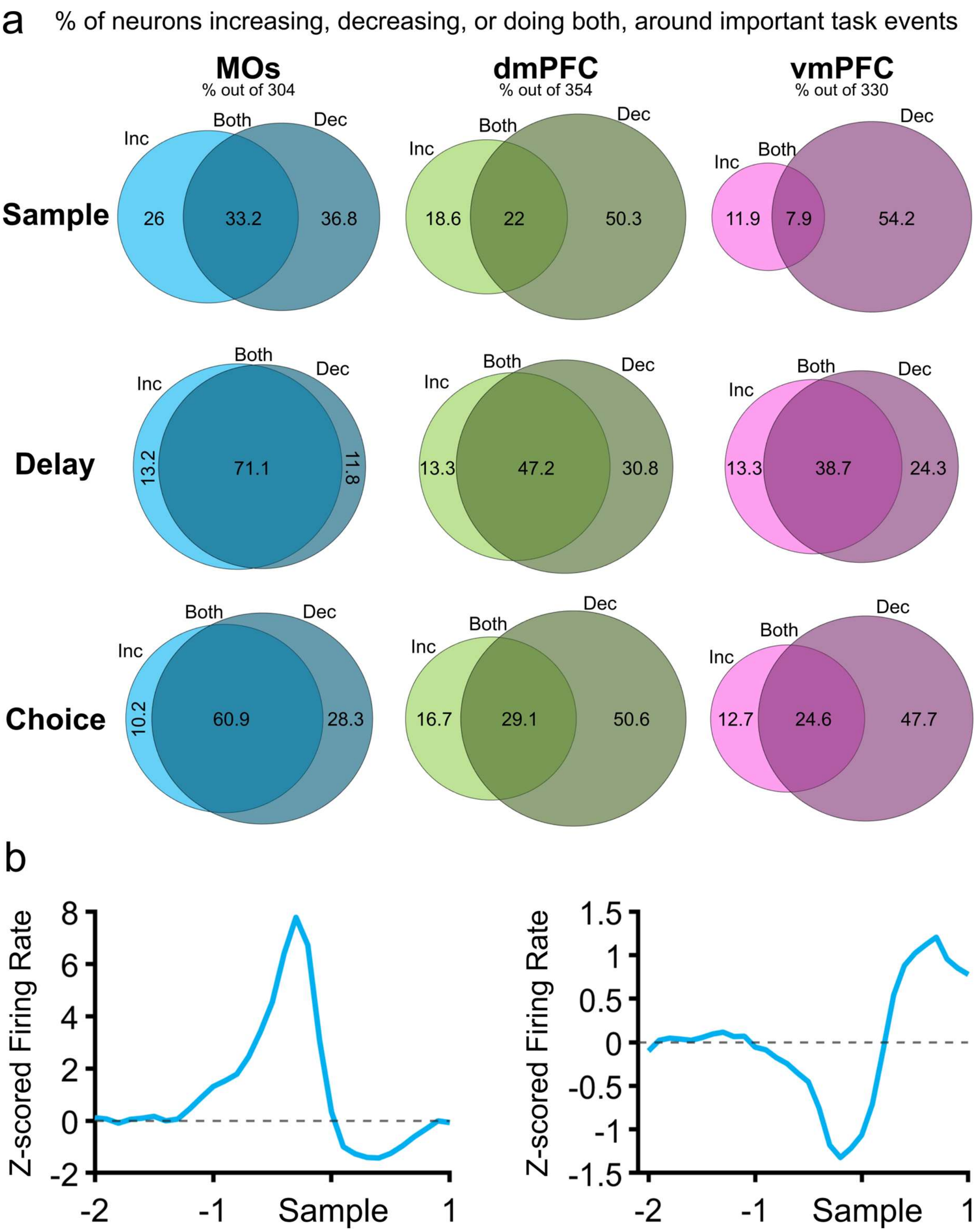

Supplementary Fig. 2: Percentage of neurons across each subregion and task event that exhibited a significant increase, decrease, or both in their Z-scored firing rate during the sample poke period. a Venn diagrams depicting the percent of neurons in each subregion and task event that exhibited a significant increase, decrease, or both in their Z-scored firing rate in at least two 100 ms time bins in a 3000-millisecond window around the indicated task phase poke. **b** Example Z-scored firing rate traces of two MOs neurons which either significantly increase and then decrease (left) or significantly decrease then increase (right) their activity around the sample poke.

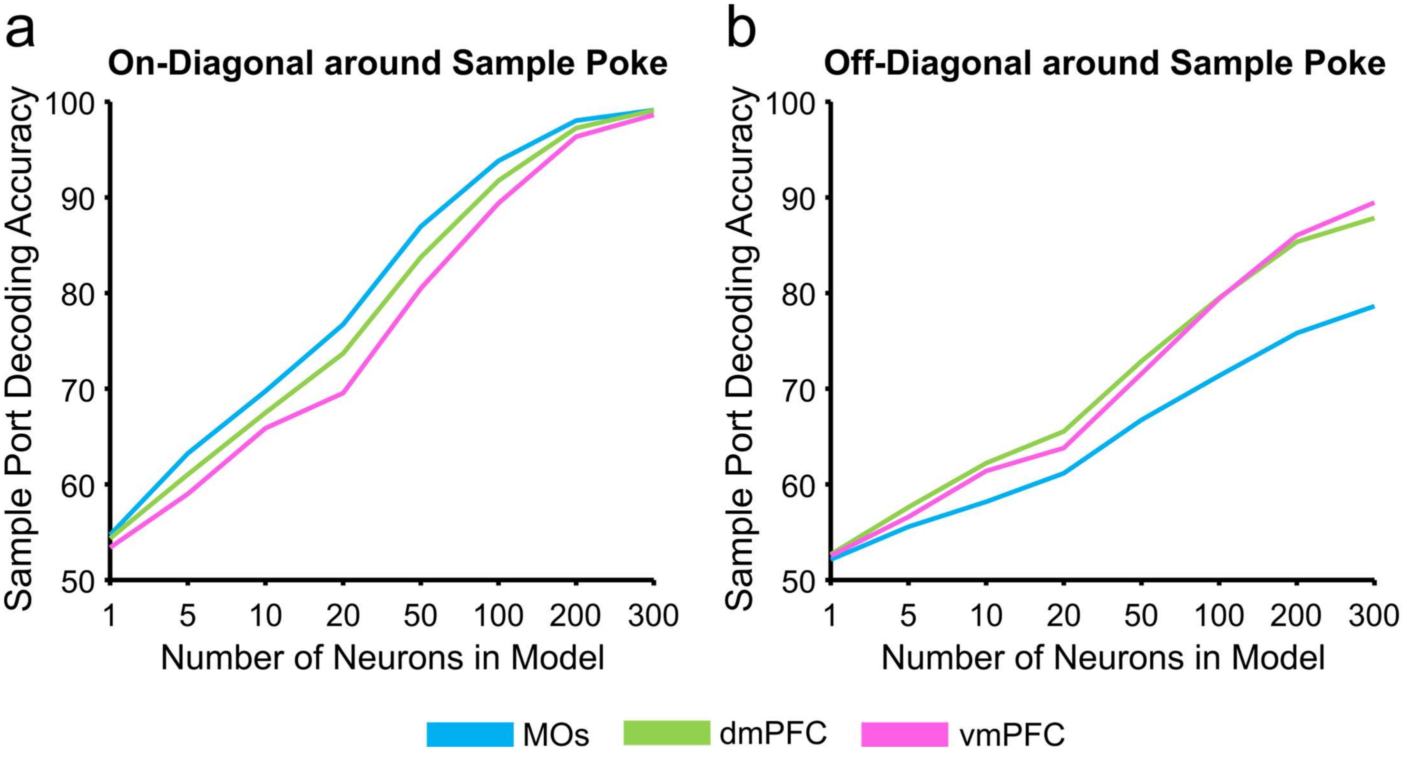

Supplementary Fig. 3: Sample location decoding accuracy around the sample poke plotted as a function of neurons used in the linear support vector machine decoding model. From -400 to +400 milliseconds around the sample poke, different numbers of neurons were randomly subsampled 100 times from each pseudopopulation, and the average sample location SVM decoding for those 100 permutations was calculated for each neuron count shown on the x-axes. **a** On-diagonal (trained and tested on the same time point) decoding of sample location around the sample poke seemed to be highest in the MOs with lower numbers of neurons, but all pseudopopulations reached ~100% decoding at 300 neurons. **b** The MOs had worse off-diagonal (trained on one time point and tested on all others, i.e. longer duration) decoding around the sample poke than both dmPFC and vmPFC at all larger neuron counts tested. Additionally, as expected, off-diagonal sample location decoding was generally lower than that of on-diagonal.

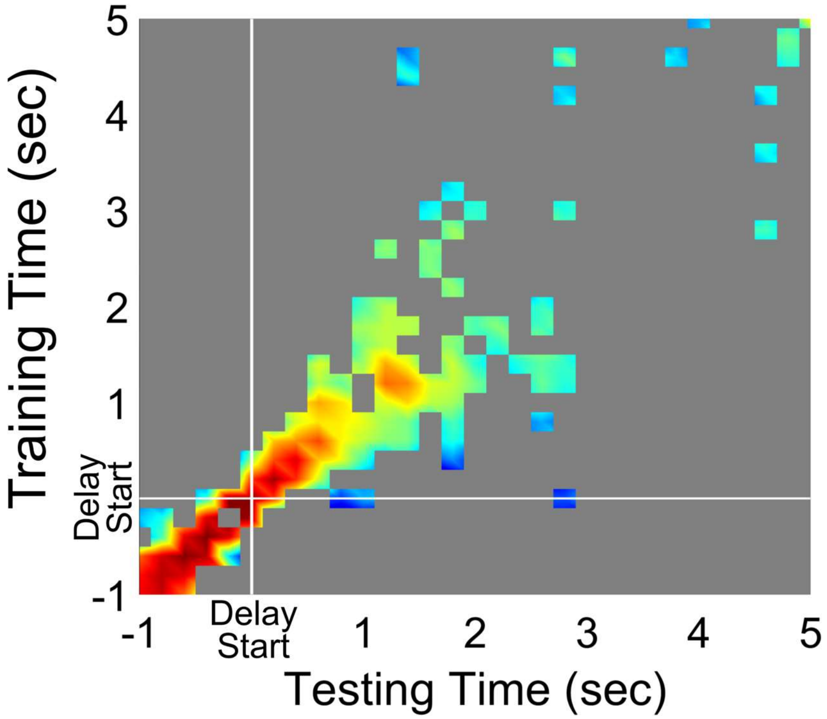

Supplementary Fig. 4: Removing the minority of dmPFC neurons with prolonged selectivity greatly reduces stable decoding accuracy. Shuffled cross-temporal linear support vector machine decoding, similar to Fig. 3g, was used to test whether removing the relatively small number of dmPFC neurons with stable sample selectivity during the delay (29 out of 354 neurons) would significantly reduce the stability of the sample location representation during the five second delay period. Stable sample selectivity was determined by finding the neurons with significantly higher firing rate in left or right sample trials in at least two total seconds (20 bins) out of five (using data from Fig. 3f).

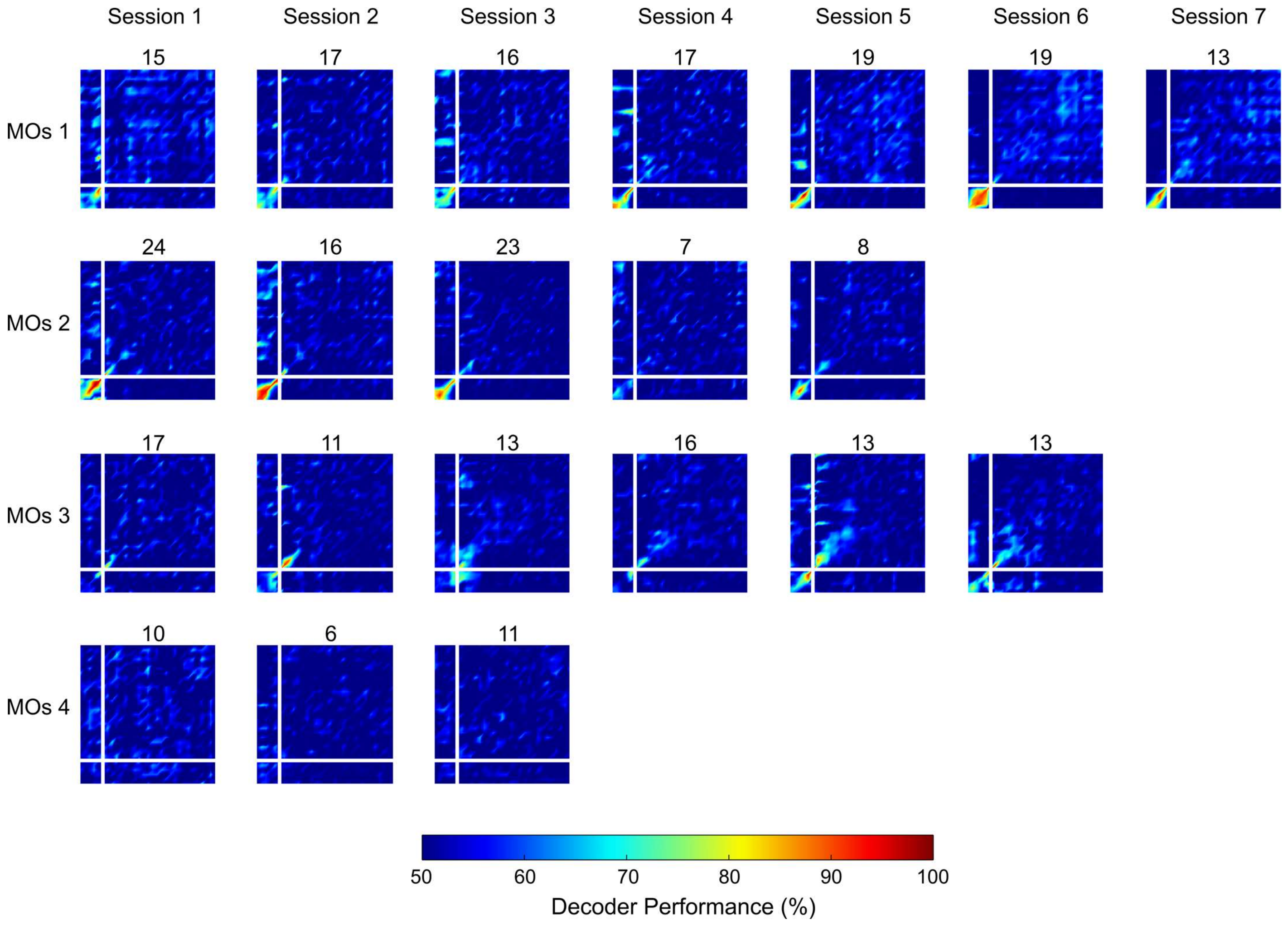

Supplementary Fig. 5: Cross-temporal support vector machine decoding of sample identity for individual sessions in the MOs during the delay period. Cross-temporal delay SVM decoding was performed on individual sessions from the MOs pseudopopulation during the delay. None of the sessions exhibit substantial cross-temporal decoding at any point during the delay period. Numbers above graphs represent the recorded neuron count for each session.

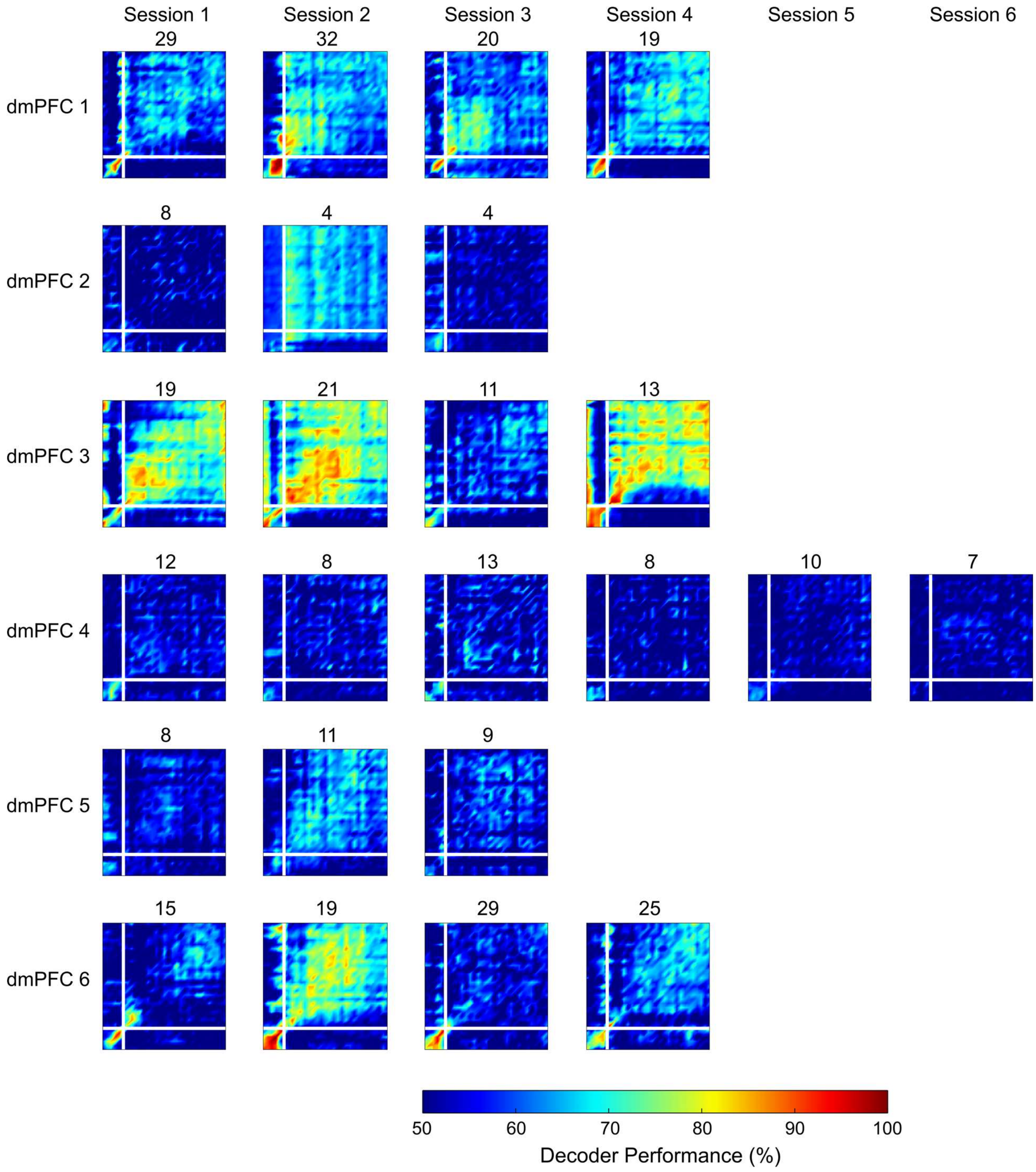

Supplementary Fig. 6: Cross-temporal support vector machine decoding of sample identity for individual sessions in the dmPFC during the delay period. Cross-temporal delay SVM decoding was performed on individual sessions from the MOs pseudopopulation during the delay. All animals except **dmPFC 4** had least one session where sample port location was decodable throughout the delay at >=70% accuracy. Interestingly, this was the same mouse we identified in Supplementary Fig. 1 which appeared to not be using working memory to complete this task based on its performance on outer versus inner choice trials. Numbers above graphs represent the recorded neuron count for each session.

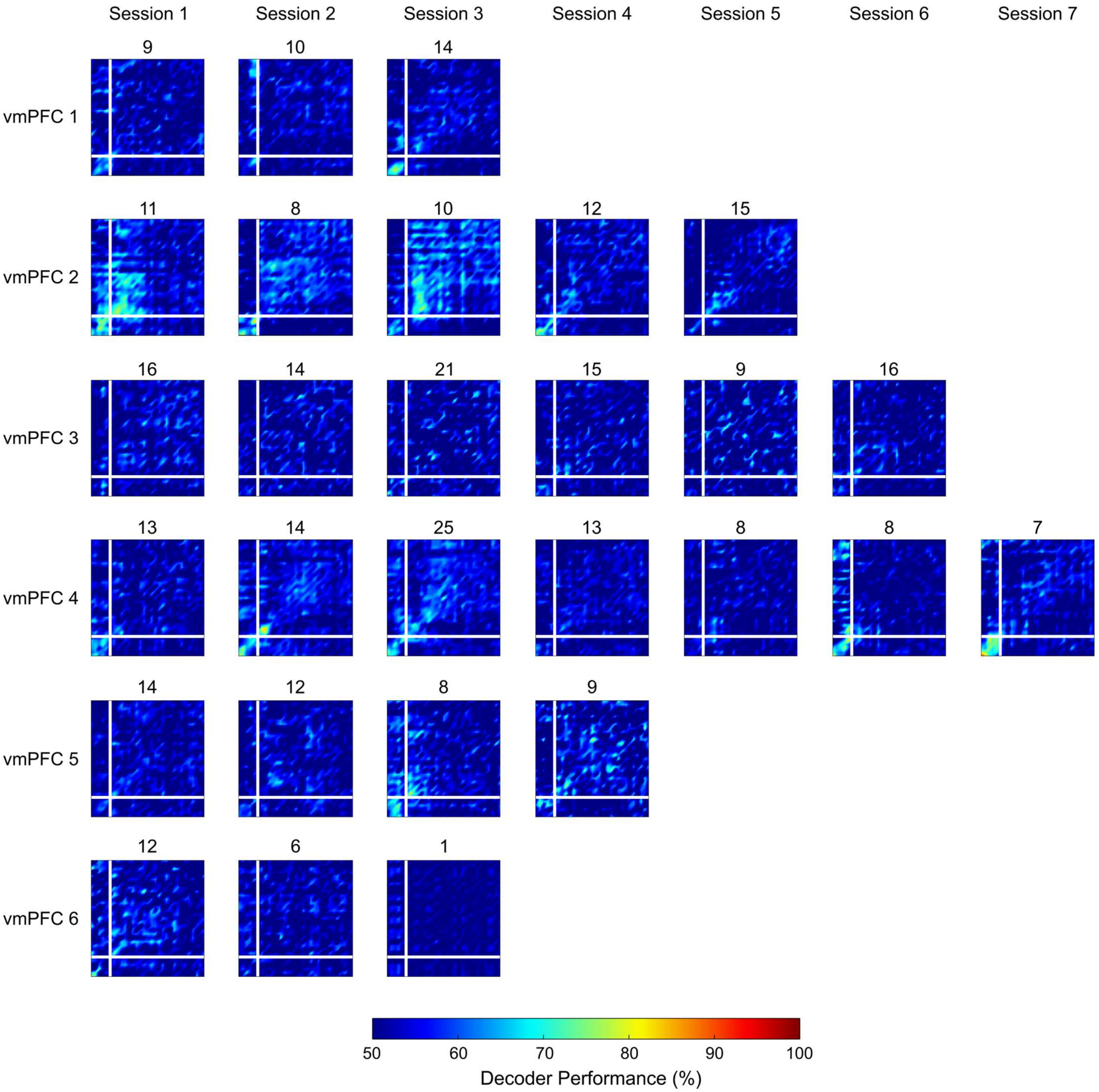

Supplementary Fig. 7: Cross-temporal support vector machine decoding of sample identity for individual sessions in the vmPFC during the delay period. Cross-temporal delay SVM decoding was performed on individual sessions from the MOs pseudopopulation during the delay. Only one recording, **vmPFC 2, Session 3**, showed any evidence of sustained sample identity decoding across the delay, although it was very weak compared to recordings from the dmPFC. Numbers above graphs represent the recorded neuron count for each session.

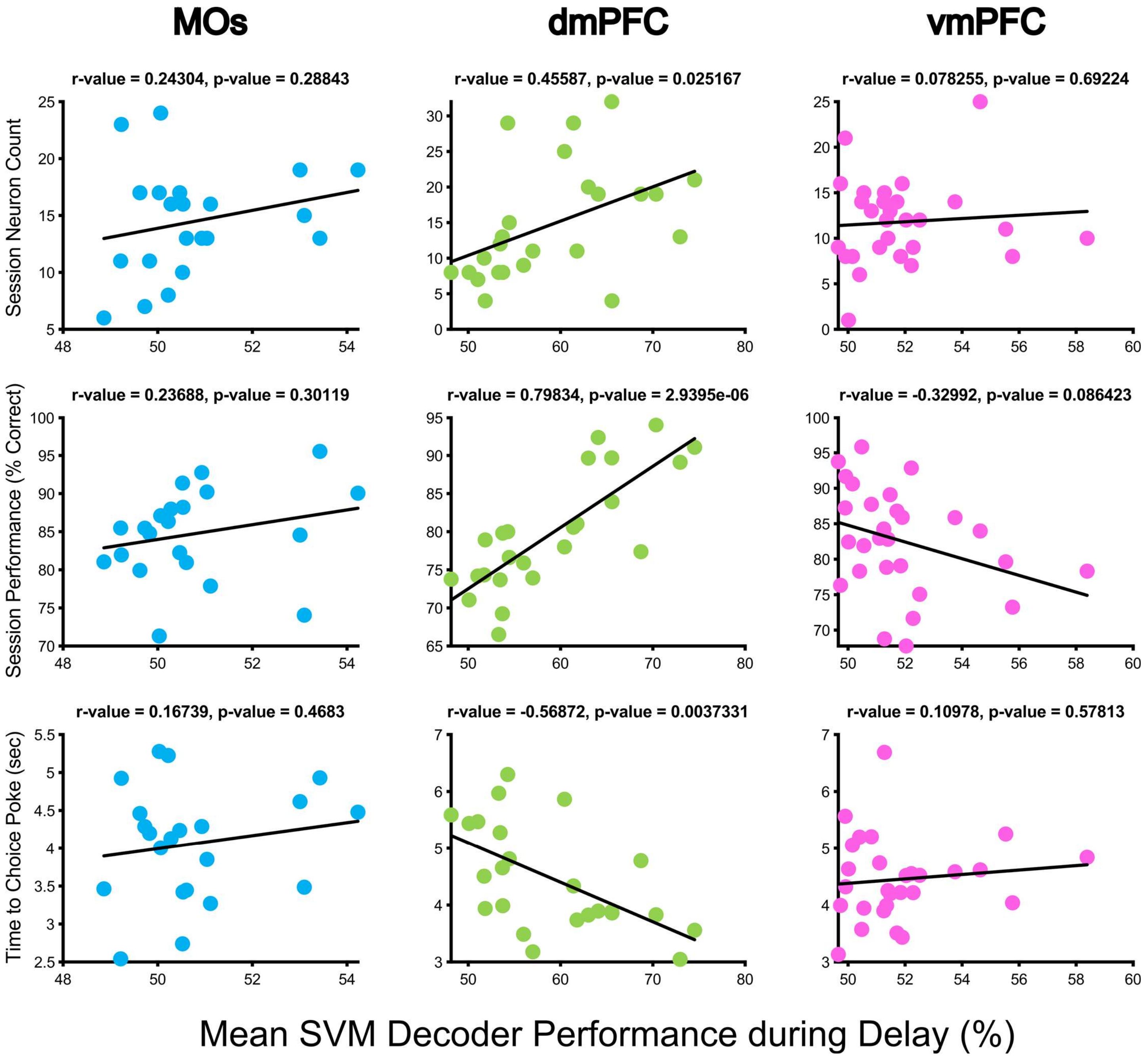

Supplementary Fig. 8: Session-based correlations between the mean delay cross-temporal SVM decoder performance and three key experimental parameters. The mean SVM decoding performance during the delay for each session was plotted against neuron count per session (top row), overall session performance (middle row), and time to choice poke from the end of the delay (bottom row). The correlation strength and significance are given above each plot. Significant correlations between SVM decoder performance and individual behavioral parameters are only found in the dmPFC (middle column). Each dot represents a different session, and therefore each column (subregion) contains the same decoder performance values across panels.

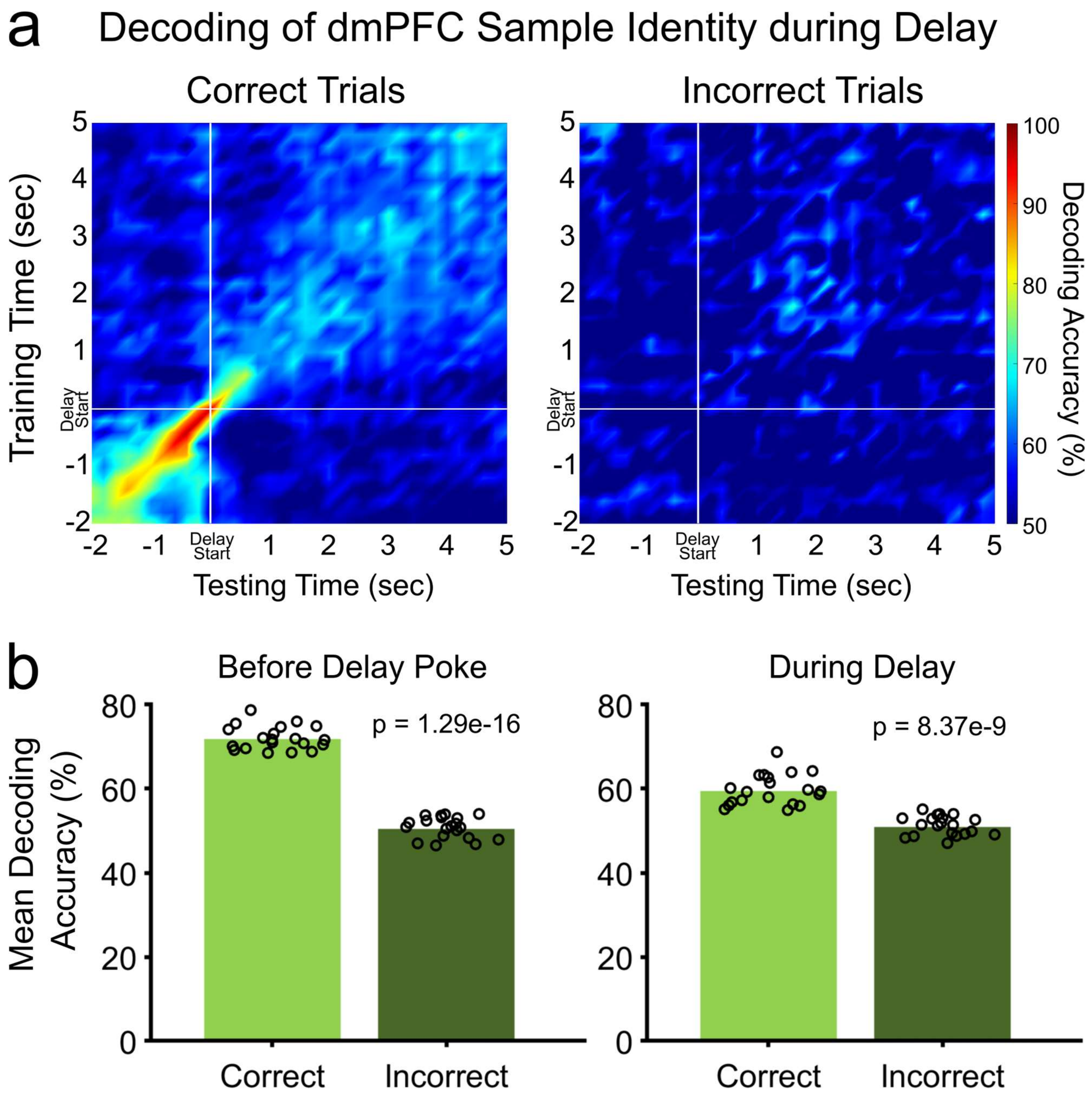

Supplementary Fig. 9: Cross-temporal support vector machine decoding of dmPFC sample identity during the delay on correct versus incorrect trials. Cross-temporal SVMs were used to compare the ability to decode sample identity on correct versus incorrect trials. Importantly, since there were sessions with few incorrect trials and some mice had mild side biases, we only used sessions with at least 5 incorrect trials when the sample was on the left and 5 incorrect trials when the sample was on the right. This reduced the number of neurons used for this analysis to 180 (from 13 sessions), about half of the initial dmPFC pseudopopulation. **a** We then subsampled 5 left and right correct and incorrect trials 20 times and took the mean decoding accuracy of these 20 subsampled SVMs at each cross-temporal bin to produce the heat maps shown. Note that since we have half the neurons and many fewer trials than for the dmPFC decoding in Fig. 3g, the overall decoding accuracy on Correct Trials is expected to be lower than that of the full pseudopopulation **b** Overall, we found significantly greater sample identity decoding using Correct Trials, both before the delay poke and during the delay (paired t-test, p-values on each plot). Each black circle on these graphs represents the mean decoding in the time window 2 s before (left), or during the 5 second delay (right) for each of the 20 subsampled SVMs.
