# Supplementary material for: Divergent subregional information processing in mouse prefrontal cortex during working memory
Source: Commun Biol. 2024 Oct 1;7:1235. doi: 10.1038/s42003-024-06926-8 (PMC11445572; doi:10.1038/s42003-024-06926-8)
Supplement: Supplementary file 3 — Reporting Summary [file 42003_2024_6926_MOESM3_ESM.pdf]

nature portfolio

Corresponding author(s): Atheir I. Abbas

Last updated by author(s): Sep 10, 2024

Reporting Summary

Nature Portfolio wishes to improve the reproducibility of the work that we publish. This form provides structure for consistency and transparency in reporting. For further information on Nature Portfolio policies, see our Editorial Policies and the Editorial Policy Checklist.

Statistics

For all statistical analyses, confirm that the following items are present in the figure legend, table legend, main text, or Methods section.

n/a	Confirmed	
☐	☒	The exact sample size (n) for each experimental group/condition, given as a discrete number and unit of measurement
☐	☒	A statement on whether measurements were taken from distinct samples or whether the same sample was measured repeatedly
☐	☒	The statistical test(s) used AND whether they are one- or two-sided<br>*Only common tests should be described solely by name; describe more complex techniques in the Methods section.*
☐	☒	A description of all covariates tested
☐	☒	A description of any assumptions or corrections, such as tests of normality and adjustment for multiple comparisons
☐	☒	A full description of the statistical parameters including central tendency (e.g. means) or other basic estimates (e.g. regression coefficient) AND variation (e.g. standard deviation) or associated estimates of uncertainty (e.g. confidence intervals)
☐	☒	For null hypothesis testing, the test statistic (e.g. F, t, r) with confidence intervals, effect sizes, degrees of freedom and P value noted<br>*Give P values as exact values whenever suitable.*
☒	☐	For Bayesian analysis, information on the choice of priors and Markov chain Monte Carlo settings
☐	☒	For hierarchical and complex designs, identification of the appropriate level for tests and full reporting of outcomes
☐	☒	Estimates of effect sizes (e.g. Cohen's d, Pearson's r), indicating how they were calculated

Our web collection on statistics for biologists contains articles on many of the points above.

Software and code

Policy information about availability of computer code

Data collection	Neural data was collected using Blackrock Central commercial software and downloaded to an attached computer. Custom, in-house MATLAB code (R2022a or later) was used to control the behavioral box and collect behavioral data.
Data analysis	Custom, in-house MATLAB code was used for all data analysis.

For manuscripts utilizing custom algorithms or software that are central to the research but not yet described in published literature, software must be made available to editors and reviewers. We strongly encourage code deposition in a community repository (e.g. GitHub). See the Nature Portfolio guidelines for submitting code & software for further information.

Data

Policy information about availability of data

All manuscripts must include a data availability statement. This statement should provide the following information, where applicable:

- Accession codes, unique identifiers, or web links for publicly available datasets
- A description of any restrictions on data availability
- For clinical datasets or third party data, please ensure that the statement adheres to our policy

Upon publication, the relevant computer code will be made publicly available on a repository as MATLAB functions.

Research involving human participants, their data, or biological material

Policy information about studies with human participants or human data. See also policy information about sex, gender (identity/presentation), and sexual orientation and race, ethnicity and racism.

Reporting on sex and gender	N/A
Reporting on race, ethnicity, or other socially relevant groupings	N/A
Population characteristics	N/A
Recruitment	N/A
Ethics oversight	N/A

Note that full information on the approval of the study protocol must also be provided in the manuscript.

Field-specific reporting

Please select the one below that is the best fit for your research. If you are not sure, read the appropriate sections before making your selection.

- [x] Life sciences
- [ ] Behavioural & social sciences
- [ ] Ecological, evolutionary & environmental sciences

For a reference copy of the document with all sections, see nature.com/documents/nr-reporting-summary-flat.pdf

Life sciences study design

All studies must disclose on these points even when the disclosure is negative.

Sample size	Sample sizes are selected such that analyses are powered to be sufficient to detect small-to-moderate sized differences in comparisons between groups.
Data exclusions	3 animals were excluded after at least one session of data was collected because the headstage fell off and we were unable to verify electrode placement as a result. Individual behavioral sessions were excluded if performance was below 65% during that session. Individual recorded neurons were excluded from analysis if the mean firing rate over the entire session was less than 0.5 Hz.
Replication	After our initial findings regarding activity differences between subregions, we collected additional data in each subregion to increase confidence in findings. We prioritized recording >300 neurons in each subregion.
Randomization	WT C57BL6 mice were randomly selected and assigned to one of the three groups of animals in this study.
Blinding	No formal blinding occurred. However, all data was collected before placements were confirmed. Furthermore, data importation and analysis are automated, and data recorded from neurons pooled as they are processed all together. This mitigates the potential impact of bias.

Reporting for specific materials, systems and methods

We require information from authors about some types of materials, experimental systems and methods used in many studies. Here, indicate whether each material, system or method listed is relevant to your study. If you are not sure if a list item applies to your research, read the appropriate section before selecting a response.

Materials & experimental systems

n/a	Involved in the study	
☒	☐	Antibodies
☒	☐	Eukaryotic cell lines
☒	☐	Palaeontology and archaeology
☐	☒	Animals and other organisms
☒	☐	Clinical data
☒	☐	Dual use research of concern
☒	☐	Plants

Methods

n/a	Involved in the study	
☒	☐	ChIP-seq
☒	☐	Flow cytometry
☒	☐	MRI-based neuroimaging

Animals and other research organisms

Policy information about studies involving animals; ARRIVE guidelines recommended for reporting animal research, and Sex and Gender in Research

Laboratory animals	C57BL6J male and female mice, approximately evenly split, age 3-4 months at the start of training. Testing completed at ages ranging from 6-9 months.
Wild animals	N/A
Reporting on sex	Both male and female animals were used. Group sizes are not sufficient to detect sex-based differences.
Field-collected samples	N/A
Ethics oversight	VA Portland Health Care System IACUC reviewed and approved the studies reported.

Note that full information on the approval of the study protocol must also be provided in the manuscript.

Plants

Seed stocks	N/A
Novel plant genotypes	N/A
Authentication	N/A
